# Treatment of Psychical Disturbances in Their First Stage

**Published:** 1854-05

**Authors:** 


From the American Medical Monthly.

*Treatment of Psychical Disturbances in their first stage.* By Dr. Erlenmeyer.

Upon the treatment of psychical disturbances at their commencement, often depends the whole course of the disease, and especially the final issue in recovery or hopeless idiocy. A very common method consists in making large abstractions of blood, which seem required by the frequent exalted temperature of the head, the accelerated circulation in the cranial arteries, and the over-distension of the veins; in a word, the cerebral congestion, as it is usually expressed. Although the experience of all countries declares this treatment inappropriate, in most cases even positively injurious—although the testimony of all our hospitals for the insane is opposed to it; yet numerous cases still occur in which patients are brought, with rapid strides, to incurable idiocy, by means of copious blood-lettings.

The time is not long gone by, when, in our best insane hospitals, the use of narcotics, in the treatment of psychical diseases, was wholly interdicted. This view was first changed by the recommendation of opium by Dr. Herman Engelken; and this remedy now began occasionally to be tried, and indeed, in somewhat larger doses than usual. The excellent result which followed this practice, in certain cases, continually encouraged to further trials; so that now it is considered indispensable by our best physicians.

The form of psychical disturbance in which opium succeeds best, is melancholy, in its various shades. It animates the patient, exalts innervation, and gives to the despairing sufferer new courage. I have tested this remedy in private practice. With few exceptions, mental disturbances, in their first stage, accost us as a melancholic temper, so that these cases also appear appropriate for the administration of opium. Upon different occasions, when I have been called to the treatment of commencing mental disturbance, I have therefore, decided upon the exhibition of opium, and have seen really surprising results from it, since many patients have not only been temporarily improved thereby, but for the most part have been completely cured.

Opium administered in large doses, operates, in many respects, entirely different from small doses. It produces no congestion of the brain; it does not induce constipation—on the contrary, I have, in several cases, observed severe diarrhœa following the use of this remedy, which required its discontinuance. I have, in all cases in which constipation followed the exhibition of small doses at the commencement, seen this disappear upon its continued and increased administration. The nutrition of the patient is very quickly increased, and I have repeatedly seen the weight of the body gain from two to three pounds a week. The courage of the patient, which in melancholy, is so depressed, becomes exalted; the constant complaints and lamantations are silenced; in short, the patient in a brief time is both corporeally and mentally changed.

In the hospitals, the exhibition of opium has been carried to six grains at a dose; and several physicians, especially those who first commended the practice, have carried it still farther, without observing any injurious effects. At the commencement of psychical disturbances, such doses, though they may be well borne, are not at once necessary; and the exhibition of from two to four grains twice a day will suffice completely to allay incipient melancholy.

The best form of opium is the powder, as such, or made into pills; whilst the tinctures and alkaloids have not been so efficient in my hands.

Whilst I now proceed to the indications and contra-indications, I should observe, in the first place, that the data brought forward are imperfect; and that I here mostly appeal to symptoms, will be excused by the reader, who knows full well that the diagnosis of the condition lying at the basis of mental maladies is infinitely difficult.

The highest indication for the exhibition of opium is the hyperæsthesia, which presents itself at the commencement of psychical disturbances in so manifold a manner. It matters not whether this hyperæsthesia be of peripheric or central origin; nor is it of any consequence in which division of nerves it occurs. The excellent effect of opium in pure neuralgias, should have long since led to its administration in hyperæsthesia of other nerves; and would certainly have done so, had not various fears, which were based more upon theory than practice, deterred therefrom. That opium is not so dangerous a remedy as it is generally represented in the manuals of Materia Medica, I have thoroughly convinced myself; and many of our German Physicians, at the head of insane hospitals, will agree with me, whose authority must be acknowledged by every one.

Almost two-thirds of all psychical maladies commence as hyperæsthesiæ. One of the most common is the hyperæsthesia of the *Nervus Vagus*, with greater or less participation of the sympathetic, in the well known form of præcordial distress, which Fleming has so well described, and which, together with headache, he enumerates as the most constant symptoms of psychical disturbances. I have observed the præcordial distress in very different constitutions, as well of central as of peripheric origin, and always perceived good effects from opium.

The result is surprising when this præcordial distress is connected with psychical hyperæthesia, a condition which is usually designated as *hypochondriacal melancholy*. These patients are fearful tormenting spirits to the physician, because they cannot be dissuaded from their hypochondriacal ideas by any process of reasoning.

A more numerous class of hyperæsthesiæ, which occur mostly at the commencement of psychical diseases, are the sensual. It is wonderful to what perversities patients are often led by this kind of alienation of the nerves of sense. A great part of the aversion to food occurring at the beginning of mental maladies, depends npon the hyperæthesia of the glossopharyngeal or olfactory nerve. In food prepared in the ordinary manner, the patients smell and taste all possible singularities; when there is also simultaneously hyperæsthesia of other nerves, often of the vagus, they are sorrowful, anxious, distrustful, smell poison in their food, which increases and justifies their anxiety, and they begin to resist nourishment. Another complaint which we frequently meet with in patients of this kind, is that those about them know their thoughts. I have found this in many cases, where there was as yet no particular mental derangement; it is evidently a minor degree of hallucination of hearing, induced by hyperæsthesia of the acoustic nerve. Such a condition very commonly precedes the outbreak of peculiar hallucinations, as I have repeatedly observed in a patient who suffers periodically from hallucinations, of hearing. A short time before the particular hallucinations, he has the sensation as if his thoughts were expressed by those about him, only that he does not clearly hear the particular words, as is the case upon the full development of the hallucination.

Most of the conditions which occur at beginning of mental diseases, may be referred to these hyperæsthesiæ, which are usually designated by all sorts of other names—*nervous irritability*, *exalted nervosity*, *nervous derangement*, *&c.*

When these hyperæsthesiæ exist in the manner just described, independent of any organic disease of the brain, manifested by anæsthesia, paralysis., &c., without the existence of any more serious affections of other important organs, of the heart, the lungs, the digestive apparatus, &c., which must be looked upon as the cause of the incipient mental disturbance, opium will do excellent service, and if it does not completely and permanently cure, it still effects an important alleviation; but in the last mentioned cases it does no good, and often may do harm.

There is also another contra-indication, which is not, however, very frequently in the way; it is vomiting occurring after the administration of small doses. We need not be much disturbed, nevertheless,, on this account, since no greater disadvantage is to be feared than that opium will do no good. I must especially insist, that a coated tongue and other gastric symptoms should not deter us from the use of opium, since this is observed in almost all cases of psychical disease, immediately at the opening of the scene, and very commonly occuring as the first expression of alienated nervous function. Opium allays these so-called gastric symptoms generally very quickly, enlivens the appetite, and stimulates nutrition better than all stomachics. There are individuals in whom there exists an idiosyncrasy against the smallest doses of this remedy, who become thereby more excited, in whom a new train of spmptoms is induced, as palpitation of the heart, ringing in the ears, greater disquiet, complete sleeplessness; in these persons we should desist at once from the farther use of opium.

Opium does excellent service, not only in melancholy, but in all other forms of psychical alteration which depend upon hyper-

æsthesia, if it is employed in the first stage of the difficulty, whilst in all psychoses of a torpid character, it produces little or no benefit.

